# Carboxy-Terminal Processing Protease Controls Production of Outer Membrane Vesicles and Biofilm in *Acinetobacter baumannii*

**DOI:** 10.3390/microorganisms9061336

**Published:** 2021-06-20

**Authors:** Rakesh Roy, Ren-In You, Chan-Hua Chang, Chiou-Ying Yang, Nien-Tsung Lin

**Affiliations:** 1 Institute of Medical Sciences, Tzu Chi University, No. 701, Sec. 3, Zhongyang Rd., Hualien 97004, Taiwan; rakeshroy1803@gmail.com; 2 Department of Laboratory Medicine and Biotechnology, Tzu Chi University, No. 701, Sec. 3, Zhongyang Rd., Hualien 97004, Taiwan; yri100@gms.tcu.edu.tw; 3 Institute of Molecular Biology, National Chung Hsing University, Taichung 40227, Taiwan; u101010424@cmu.edu.tw; 4 Department of Microbiology, School of Medicine, Tzu Chi University, No. 701, Sec. 3, Zhongyang Rd., Hualien 97004, Taiwan

**Keywords:** *Acinetobacter baumannii*, carboxy-terminal processing protease (Ctp), outer-membrane vesicles (OMVs), extracellular DNA (eDNA), capsular polysaccharides (CPSs)

## Abstract

Carboxy-terminal processing protease (Ctp) is a serine protease that controls multiple cellular processes through posttranslational modification of proteins. *Acinetobacter baumannii* ATCC 17978 *ctp* mutant, namely MR14, is known to cause cell wall defects and autolysis. The objective of this study was to investigate the role of *ctp* mutation–driven autolysis in regulating biofilms in *A. baumannii* and to evaluate the vesiculation caused by cell wall defects. We found that in *A. baumannii*, Ctp is localized in the cytoplasmic membrane, and loss of Ctp function enhances the biofilm-forming ability of *A. baumannii*. Quantification of the matrix components revealed that extracellular DNA (eDNA) and proteins were the chief constituents of MR14 biofilm, and the transmission electron microscopy further indicated the presence of numerous dead cells compared with ATCC 17978. The large number of MR14 dead cells is potentially the result of compromised outer membrane integrity, as demonstrated by its high sensitivity to sodium dodecyl sulfate (SDS) and ethylenediaminetetraacetic acid (EDTA). MR14 also exhibited the hypervesiculation phenotype, producing outer-membrane vesicles (OMVs) of large mean size. The MR14 OMVs were more cytotoxic toward A549 cells than ATCC 17978 OMVs. Our overall results indicate that *A. baumannii* *ctp* negatively controls pathogenic traits through autolysis and OMV biogenesis.

## 1. Introduction

*Acinetobacter baumannii*, an opportunistic, nosocomial pathogen, is associated with an outbreak of various human infections related to wound, burn, bloodstream, urinary tract and pneumonia, leading to increasing mortality [1]. Due to an increase in the prevalence of multidrug-resistant *A. baumannii*, it is considered a priority pathogen in the research and development of novel antimicrobials. The bacterial cell wall as a therapeutic target has been a topic of research for decades because of its essentiality [2]. However, mechanistic studies targeting the cell wall of *A. baumannii* are limited.

The major component of the cell wall is peptidoglycan (PG). The precursors of PG are assembled and inserted into existing PG in the presence of enzymes collectively known as penicillin-binding proteins (PBPs) [3]. PBPs play an essential role in cell wall elongation, shape determination, septation and PG cross-linking [4]. A study demonstrated that PBP3 and PBP7 are processed by Prc protease in *Escherichia coli* [5]. In addition, in *Salmonella enterica prc* mutants, altered activity of PBP3, PBP4, PBP5/6 and PBP7 was reported [6]. The *prc* gene is also known as *tsp* and is homologous to *ctpA* [7]. Its gene product has been classified in MEROPS as a serine protease belonging to the S41 family [8]. In *Borrelia burgdorferi*, Prc protease processes outer membrane (OM) proteins P13 and BB0323 [9]. In addition, in *Pseudomonas aeruginosa,* the CtpA forms a complex with LbcA to cleave four substrates, namely MepM, PA1198, PA1199 and PA440, all of which are predicted to play a role in PG biogenesis [10]. In *E. coli*, NlpI was identified as an adapter molecule required to enhance the stability and activity of Prc [11]. The Prc-NlpI complex then regulates the cell wall cross-link hydrolase MepS (formerly Spr) at the cellular level to facilitate the proper growth and enlargement of the PG sacculus [12]. By contrast, a recent study reported MltG to be the substrate of Prc and demonstrated that NlpI does not play the role of an adapter in the interaction between Prc and MltG, an inner membrane–associated lytic transglycosylase involved in the biogenesis of PG [13]. Interestingly, most substrates of Prc have been found to be involved in PG biosynthesis.

When the PG cell wall is compromised, the bacterial cell becomes susceptible to osmotic lysis, which may cause the cell to burst. The *ctpA* mutant in *Rhizobium leguminosarum* bv. *viciae* 3841 exhibited a compromised and detached OM [14]. The overexpression of MltG, a substrate of Prc, results in morphological defects and cell lysis [13]. A study reported that the biogenesis of outer-membrane vesicles (OMVs) is a consequence of explosive cell lysis in *P. aeruginosa*, probably caused by rapid loss of the structural integrity of cell wall PG [15]. OMVs are spherical membrane bilayer structures that are released from the OM of gram-negative bacteria. The diameter of OMVs ranges from 50 to 250 nm [16]. They may be produced as a defense mechanism against antimicrobial peptides [17], antibiotics [18] or phages [19] or in response to stress caused by ultraviolet radiation [20] or a cationic surfactant [21]. Several studies have shown that the OMVs produced by bacteria have diverse functions including cell–cell communication, pathogenesis, immunomodulation, surface modification and in vivo adaptation [22,23,24]. Mutation in NlpI, an adapter molecule for CtpA, results in reduced fitness and envelope integrity, thereby increasing the amount of OMV formation [25]. In addition, the *nlpI* mutant displayed a significant decrease in OM-PG stabilizing cross-links and PG cross-linked lipoprotein levels, which may have contributed to the hypervesiculation phenotype [26]. Moreover, the *nlpI* mutant was shown to increase the extracellular DNA (eDNA) level and cause cell lysis in *E. coli* [27], and complementation of the *nlpI* gene led to suppression of the biofilm phenotype of the *nlpI* mutant in *S. enterica* Serovar Typhimurium [28]. The *P. aeruginosa ctpA* mutant was also reported to attach robustly to glass surfaces [10]. eDNA, which is an essential constituent of biofilms, is secreted through active secretion or cell lysis [29]. Autolysis-dependent eDNA release has been shown to promote biofilm formation in several bacterial species [30,31,32]. Thus, the aforementioned studies highlight the importance of PG remodeling and autolysis in regulating OMV biogenesis and biofilm formation.

In our previous study, we performed random Tn*10* transposon mutagenesis of *A. baumannii* ATCC 17978, extracted a *ctp* mutant, MR14 and generated a complementation strain, MR14C and found that MR14 exhibited envelope defects and autolysis in the late exponential phase, displayed significantly increased levels of eDNA, cell surface hydrophobicity and attenuated virulence compared with the parental strain ATCC 17978 [33]. A comparison of the phenotypes of MR14 reported by previous studies on Ctp/Prc and its substrates led us to hypothesize that this strain has high biofilm formation and OMV biogenesis abilities. The dysregulation of PG remodeling caused by *ctp* gene mutation could weaken the interaction between PG and OM, resulting in blebbing of the OM as proposed previously [34]. According to another hypothesis model of OMV biogenesis and considering the role of Ctp in cleaving incorrectly folded proteins in the periplasm [10], accumulation of misfolded proteins within the periplasm of MR14 could result in increased OMV biogenesis [35]. Hence, this study investigated the correlation between *ctp* gene mutation in *A. baumannii* and biofilm formation and OMV biogenesis abilities. While in our previous paper we demonstrated *ctp* gene in *A. baumannii* to have role in motility, membrane integrity, autolysis and virulence [33], in our current paper we studied about the consequences of *ctp* gene mutation in terms of biofilm and OMV biogenesis that may be the indirect effect of enhanced membrane defects and elevated cell envelope stress responses caused by the loss of Ctp. We also checked for the possible vital components required for biofilm formation and investigated whether hypervesiculation caused by *ctp* gene mutation have role in virulence.

## 2. Materials and Methods

### 2.1. Bacterial Strains, Media and Culture Conditions

The bacterial strains used in this study were wild-type *A. baumannii* ATCC 17978; a transposon-mediated nonmotile derivative strain, MR14; and a complementation strain, MR14C, as described in our previous paper [33]. *E. coli* TOP10 (Invitrogen Life Technologies, Carlsbad, CA, USA) was used as a cloning host. We used pTZ57R/T (Fermentas, Waltham, MA, USA) for cloning polymerase chain reaction (PCR) products in accordance with the manufacturer’s instructions. The shuttle vector pABCL_llb was used to generate pABCL_llb:0493, which was then electroporated into MR14 to generate MR14C. Vector pET-30c (Novagen, Madison, WI, USA) was used to generate pET-30c_0493, which was then transformed into *E. coli* strain BL21(DE3) (TransGen Biotech, Beijing, China) to induce and overexpress Ctp. All strains were grown in Luria-Bertani broth (LB) at 37 °C under vigorous shaking (150 rpm). Antibiotics were used at the following concentrations: ampicillin, 100 µg/mL; chloramphenicol, 30 µg/mL; apramycin, 50 µg/mL; and kanamycin, 50 µg/mL. A biofilm assay was performed using a KIMBLE Plain Disposable Borosilicate Glass Tube (13 × 100 mm^2^, 10 mL) and a 24-well polypropylene plate.

### 2.2. Molecular Cloning, Expression and Purification of Ctp

The A1S_0493 loci of *A. baumannii* ATCC 17978 was amplified from its genomic DNA through PCR with the primers *BamH*I_0493F (GGATCCTGCGAACACATCTAACCGTA) and *Sal*l_0493R (GTCGACTGACACTTTGATGATAAATGC) that resulted in the generation of 2081 bp PCR fragment. The underlined nucleotides indicate restriction sites, namely *BamH*l and *Sal*l, present on the 5′ end of the forward and reverse primers, respectively. The PCR fragment was then cloned into TA cloning vector pTZ57R/T (Fermentas, Waltham, MA, USA) in accordance with the manufacturer’s instructions to obtain pTZ57R/T_0493, which was then sequenced to confirm loci A1S_0493. After confirmation, the vector was isolated using the Plasmid Mini Kit (Qiagen, Valencia, CA, USA), digested using restriction enzymes *BamH*l and *Sal*l, and ligated into the pET-30c expression vector digested with the same enzymes as that for pTZ57R/T_0493. The resulting pET-30c_0493 plasmids were transformed into *E. coli* BL21 (DE3) cells. The cells were then cultured in LB overnight, subcultured 1:100 in fresh LB medium and grown until an OD_600_ of approximately 0.6 was achieved. Recombinant N-terminal 6xHis-tagged Ctp expression was then induced using 0.5 mM isopropyl-β-d-thiogalactopyranoside (IPTG), and the cells were further grown for 2.5 h at 37 °C. The cells were then harvested through centrifugation at 8000× *g* at 4 °C for 15 min. The obtained pellets were resuspended in lysis buffer (50 mM NaH_2_PO_4_ and 0.3 M NaCl; pH 8.0) and sonicated, after which the overexpressed protein was purified from the insoluble fraction through affinity chromatography with a Ni–nitrilotriacetic acid (NTA) column (Qiagen) by using 8 M urea. The proteins obtained after elution were dialyzed and stored at −20 °C) until use.

### 2.3. Polyclonal Antibody Production

Following overexpression and purification of Ctp, the protein samples were sent to Protech Technology Enterprise Co., Ltd. (Taipei, Taiwan) for polyclonal antibody production. The polyclonal antibodies were generated in rabbits.

### 2.4. Subcellular Localization of Ctp

We performed membrane fractionation as per the protocol described previously [36,37] with some modifications. In brief, overnight cultures of ATCC 17978 were refreshed into fresh LB to obtain an initial OD_600_ = 0.01. The cultures were then grown to the mid-log phase. Cells were then harvested through centrifugation at 8000× *g* for 15 min. Thereafter, the cells were washed three times and resuspended in periplasting buffer containing 20% sucrose, 1mM EDTA and 30,000 U of freshly prepared lysozyme, incubated on ice with stirring for 10 min. It was then centrifuged, resuspended the pellet in 10 mM magnesium chloride and incubated at 30 °C for 5 min followed by incubation on ice for 10 min. The sample was freeze-thawed and then centrifuged at 8000× *g* for 15 min at 4 °C. The cells were then broken by passage through the Constant Systems CF1 Cell Disrupter (Constant Systems Ltd., Low March, Daventry, Northamptonshire, UK) at 30 kpsi and the lysate was spun down at 11,000× *g* for 30 min at 4 °C to remove un-lysed cells. The supernatant containing membrane fraction was ultracentrifuged using Ti45 Beckman rotor at 130,000× *g* for 1.5 h at 4 °C. The pellet containing total membrane was then resuspended in low-density isopycnic-sucrose gradient solution (20% sucrose, 1 mM EDTA, 1 mM Tris pH 7.5) and layered carefully over 73%-45%-20% sucrose gradient as described previously [37]. 73%-45%-20% represents high-density, medium-density and low-density isopycnic-sucrose gradient solution, respectively. It was then ultracentrifuged at 288,000× *g* for 18 h at 4 °C. The layer of proteins formed at the interface between 20%–45% and 45%–73% sucrose was collected as inner membrane and OM, respectively. To limit the potential mixing of membranes, we first collected inner membrane fraction from the top using P1000 pipette tip and then OM fraction was obtained by puncturing the centrifuge tubes with the help of a needle. Each inner and outer membrane sample were then centrifuged at 184,500× *g* for 1 h at 4 °C and the pellets were resuspended using membrane storage buffer (10 mM Tris pH 7.5) and kept at −20 °C until use. The protein concentrations in each fraction were determined using the bicinchoninic acid (BCA) assay (Pierce BCA protein assay kit, Thermo Fisher Scientific, Rockford, IL, USA) in accordance with the manufacturer’s instructions. Samples were analyzed through Western blotting by using goat antirabbit IgG-AP conjugate as a secondary antibody at 3000× dilution. The purity of inner and outer membrane fraction was verified using antibody against GP2 and OmpA, respectively. GP2 protein is a membrane associated *A. baumannii* glycoprotein identified previously [38]. The sequence prediction revealed that it is an inner membrane protein and subsequently confirmed by Western blot using GP2 specific antibodies produced by immunization of mice with *E. coli* produced recombinant protein [39].

### 2.5. Capsular Polysaccharide Visualization and Quantification

We first visualized capsular polysaccharides (CPSs) using Congo Red (CR) as per the protocol described previously [40]. Briefly, ATCC 17978, MR14 and MR14C strains were grown in LB containing CR (40 µg/mL) for 24 h at 37 °C; this was followed by visualization of slime at the air–liquid interface on the glass surface and inspection of CR-stained aggregates at the bottom of the cultures. Overnight cultures were also aliquoted to approximately 10^9^ cells and centrifuged at 7500× *g* to obtain a pellet. We then resuspended the pellet in 1 mL of LB containing 40 µg/mL CR and incubated the mixture for 15 min at RT, followed by centrifugation at 7500× *g* to evaluate the ability of the pellet to bind to CR. Furthermore, scanning electron microscopy (SEM) was used to visualize the CPSs formed by MR14 and to compared them with those formed by the ATCC 17978 and MR14C strains. For this purpose, 13-mm-diameter plastic coverslips (Thermanox Plastic coverslips, Nunc TM Brand product, Rochester, NY, USA) were used. The biofilm formed on the surface was rinsed twice with 0.8% saline using the protocol described in our previous paper [33] and visualized through SEM (Hitachi, S-4700, Tokyo, Japan).

The CPS extraction and quantification were performed as described previously [41,42] with some modifications. Briefly, overnight bacterial cultures were subcultured 1:100 in fresh LB medium and grown till OD_600_ of approximately 0.6 was achieved. The cultures were then diluted to OD_600_ = 0.1 in 20 mL of LB and cultured in a shaking incubator at 37 °C for 24 h. Thereafter, to 12 mL of culture, 8 mL of distilled water was added, with the mixture then vortexed, centrifuged at 7500× *g* for 20 min followed by carefully separation of the supernatant. To the supernatant, trichloroacetic acid was added to achieve a final concentration of 14%. The solution was then homogenized to denature the protein by using a shaker for 30–40 min at 90 rpm and through centrifugation at 7500× *g* for 20 min at 4 °C. The pellet was discarded and prechilled ethanol (95%) and NaCl were added to obtain final concentrations 70% and 40 mM, respectively. The mixtures were then mixed evenly and stored at −20 °C overnight, after which they were centrifuged at 14,000× *g* for 20 min, with the resultant pellet then air dried. Finally, the pellet was dissolved in 1 mL of distilled water, and 40 µL of this solution was transferred to 96-well plates. To 40 µL of the solution, 100 µL of anthrone reagent was added, and the mixture was then incubated at 95 °C in a water bath for 3 min and incubated at RT for 5 min. Thereafter, it was placed in a 45 °C water bath for 15 min, and the OD_630_ was measured using an enzyme-linked immunosorbent assay (ELISA) reader (Multiskan^®^ Spectrum, Thermo Fisher Scientific Oy, Vantaa, Finland).

### 2.6. Biofilm Assay

Biofilm formation assays were performed through crystal violet (CV) staining as described previously [43] with some modifications. ATCC 17978, MR14 and MR14C strains were cultured in LB for overnight. The strains were then refreshed 1:100 in fresh LB medium and grown until an OD_600_ of approximately 0.6 was achieved. The cultures were then diluted to OD_600_ = 0.01 in 1 mL of fresh LB within disposable borosilicate glass tubes. The tubes were incubated at 37 °C for 24 h without shaking. The tubes containing biofilm were washed twice with 0.8% saline and dried for 5 min at RT. The biofilm was then stained with 0.1% CV for 30 min, washed twice with 0.8% saline and then air dried for 15 min. Finally, the CV-stained biofilm was completely dissolved using 95% ethanol, and OD_570_ was measured. LB alone was used as a negative control to reduce background staining. The test was performed three times independently.

### 2.7. Quantification of the Biofilm Components

The biofilm components—namely, eDNA, protein and polysaccharides—Were quantified following a previously described protocol [44], with some modifications. In brief, biofilm was allowed to form in disposable borosilicate glass tubes as described in Section 2.6. The planktonic cells were discarded, and the wells were washed twice with 0.8% saline. The biofilm matrix was then solubilized in 500 µL of 0.8% saline. The biofilm matrixes from 10 wells were pooled together. To half of the pooled sample, sodium dodecyl sulfate (SDS) at 0.01% concentration was added, which was followed by incubation at RT for 4 h at 150 rpm, centrifugation to remove cell debris and filtration through a 0.22 µm syringe filter. The filtered solution was then used to quantify the eDNA and protein. The other half of the sample (homogenate) was used for polysaccharide quantification, determination of the biofilm viable cell count and transmission electron microscopy (TEM) analysis.

The amount of eDNA within the biofilm matrix was quantified by measuring the fluorescence of propidium iodide (PI)-bound eDNA as per the protocol described previously [45,46], with some modifications. In brief, 100 µL of the filtrate was added to 100 µL of PI (5 µM) dissolved in 0.8% saline. The sample was incubated at RT for 15 min, after which the fluorescence of PI-bound eDNA at the excitation/emission wavelengths 535/620 nm was measured using a microplate reader. The BCA assay (Pierce BCA protein assay kit, Thermo Fisher Scientific, Rockford, IL, USA) was used to quantify proteins within the biofilm matrix with BSA as a standard and by following the manufacturer’s protocol. The amount of eDNA and protein in the biofilm matrix was quantified and normalized to OD_600_.

The polysaccharides of the biofilm matrix were quantified using CR. In brief, to 1 mL of the homogenate obtained from the biofilm matrix, CR was added to make up a final concentration of 40 µg/mL. The solution was then incubated with shaking at 37 °C for 2 h. Finally, it was centrifuged at 7500× *g* for 5 min, and the supernatant’s absorbance was measured at 490 nm. Saline with only CR was used as a control. The percentage of CR-bound cells was measured using the following equation: % of CR-bound cells = (OD_490_ of control − OD_490_ of homogenate’s supernatant)/OD_490_ of control × 100%. We also quantified the viable cells within the biofilm matrix by vortexing of the matrix homogenate sample, which was followed by sonication in a bath sonicator (Branson, North Olmstead, OH, USA) for 5 min, and then making a serial dilution of 100 µL, spreading it over an LB agar plate, incubating it overnight at 37 °C and counting the colony forming units (CFUs).

### 2.8. Biofilm Inhibition and Disruption Assay

After determining the biofilm matrix components contributing to biofilm formation in MR14, we evaluated the biofilm-forming ability of this strain in the absence of those components. For this purpose, we used DNase and Proteinase K to degrade the eDNA and protein components of the biofilm by following the previously described protocol with some modifications [44]. First, we determined the role of eDNA in biofilm formation through a biofilm inhibition assay. In brief, DNase (2 U/mL) was added to the culture at time 0. The biofilm-forming assay was then performed as described in Section 2.6, and the biofilm-forming ability of these strains was compared with that in the absence of DNase. We also conducted a biofilm disruption assay in which the biofilm was allowed to form as described in Section 2.6 for 24 h at 37 °C without shaking. After 24 h, planktonic cells were discarded. The biofilm matrix was then washed twice with 0.8% saline, and 5 U/mL DNase and 100 µg/mL Proteinase K enzymes were added separately followed by further incubation at 37 °C for 12 h. After 12 h, the solutions were decanted, the biofilm matrix was washed twice with 0.8% saline, and the biofilm was quantified using CV assay. All the experiments were performed in triplicate, and a paired *t* test was used to determine the decrease in biofilm matrix due to the addition of DNase and Proteinase K.

### 2.9. TEM Analysis of Biofilm and Planktonic Cells

For the TEM analysis of ATCC 17978 and MR14 within the biofilm, 1 mL of the biofilm matrix homogenate was centrifuged at 2000× *g*, and the pellet was resuspended in 100 µL of ddH_2_O. Thereafter, 10 µL of the sample was placed on a grid, soaked with filter paper, stained with 10 µL of uranyl acetate, and viewed through TEM (H-7500, Hitachi High-technologies, Tokyo, Japan). We visualized planktonic cells too following the same protocol as mentioned above.

### 2.10. SDS and EDTA Sensitivity Assay

The sensitivity of ATCC 17978 and MR14 strains to SDS and EDTA was analyzed as previously described [47], with some modifications. In brief, overnight cultures of the bacterial strains were subcultured 1:50 in fresh LB for 1 h. The cultures were then diluted again to obtain initial OD_600_ = 0.01 in tubes containing SDS (0.01%), EDTA (0.1 mM) and SDS/EDTA, separately. The tubes were then incubated at 37 °C with shaking for 8 h. After 8 h, the planktonic growth at OD_600_ was measured and compared with that in the corresponding untreated group. We also evaluated the sensitivity of ATCC 17978, MR14 and MR14C strains at different EDTA concentrations. To achieve this, we subcultured overnight cultures of bacterial strains as above and diluted these strains to obtain initial OD_600_ = 0.01 in fresh LB medium containing EDTA at concentrations 0–1 mM and visually observed growth defects after 8 h on the basis of the turbidity. We then selected 0.4 mM EDTA to quantify growth defects at OD_600_. Experiments were performed in at least three biological replicates.

### 2.11. OMV Isolation, Visualization and Quantification

First, overnight cultures of ATCC 17978, MR14 and MR14C strains were subcultured 1:100 in fresh LB till an OD_600_ of approximately 0.6 is achieved and diluted them to acquire initial OD_600_ = 0.01. The cultures were then incubated at 37 °C with vigorous shaking (150 rpm). Thereafter, 1 mL of culture was collected at 2, 5, 12 and 18 h to evaluate the presence of OMVs. This was achieved through centrifugation at 2000× *g* and resuspending the pellet in ddH_2_O. Then, 10 µL of the sample was placed on a grid and visualized under TEM.

To validate increased OMV visualization around MR14 cells, we isolated OMVs from culture supernatants as per the protocol described previously [48], with some modifications. Briefly, overnight cultures were refreshed in 500 mL of fresh LB to achieve initial OD_600_ = 0.01 as described above. The culture was then incubated at 37 °C for 15 h with vigorous shaking. The bacterial cells were separated from the supernatant through centrifugation at 8000× *g* for 15 min. The supernatant was passed through a 0.22-µm syringe filter to remove residual cells. After filtration, the supernatant was concentrated using the Amicon Stirred Cells pressure-based sample concentrator by using a 100-kDa filter membrane. The concentrated sample was again passed through a 0.22-µm syringe filter, and this was followed by ultracentrifugation at 150,000× *g* for 3 h at 4 °C. The OMV fraction was washed and resuspended in 0.8% saline. To ensure that the OMV sample was free from viable cells, we poured 10 µL of the OMV sample onto an agar plate and incubated it at 37 °C overnight. The OMV samples were then viewed through TEM. OMV samples were quantified on the basis of the protein concentration of OMVs determined using a BCA assay (Pierce BCA protein assay kit) as per the manufacturer’s protocol. Same volumes of OMVs (15 µL) isolated from ATCC 17978 and MR14 were mixed with SDS loading buffer and heated at 100 °C for 10 min, and this was followed by sample separation using 10% SDS-polyacrylamide gel electrophoresis (PAGE). We also measured the fluorescence obtained from DNA-bound SYTO9 to detect and quantify OMV-associated internal and external DNA. Briefly, 100 µL of OMVs from ATCC 17978 and MR14 each containing 5 µg of protein was added to 100 µL of SYTO9 (5 µM) in 0.8% saline and incubated for 15 min; the fluorescence of SYTO9-bound DNA was then measured at excitation/emission wavelengths of 485/528 nm by using a microplate reader.

### 2.12. Nanoparticle Tracking Analysis of OMVs

The size and concentration of the OMVs were determined using a NanoSight NS300 instrument (Malvern Ltd.) as per the protocol described previously [49], with some modifications. In brief, OMV samples from ATCC 17978 and MR14 at the protein concentration of 1 mg/mL were prepared. They were then diluted to 1:1000 in 0.9% saline and loaded into the sample chamber. Three individual 60-s videos were recorded for each sample, and size distribution histograms were prepared using NanoSight software NTA 3.4 Build 3.4.003. The experiments were performed at RT.

### 2.13. Cell Viability Test

The cytotoxic effect of the OMVs was examined using tetrazolium-based MTT assays as previous described [50], with some modifications. Briefly, human lung epithelial A549 cells (5 × 10^3^ cells/well) were incubated for 20 h with various concentrations of OMVs (0–50 μg/mL) isolated from ATCC 17978 and MR14. After stimulation with OMVs, the cells were incubated with MTT for 4 h at 37 °C, and formazan was dissolved using dimethyl sulfoxide. The absorbance of cell pellets was then read using an ELISA reader (Varioskan^®^ Flash, Thermo Fisher Scientific Oy, Vantaa, Finland) at 570 nm. The assay was performed in biological triplicate, and the OD_570_ was compared with that of the nontreated control group.

### 2.14. Statistical Analyses

Each assay was performed in triplicate, and the results are represented as mean ± standard deviation (SD). The Student’s *t* test and analysis of variance followed by Tukey’s multiple comparison test were performed using GraphPad Prism version 8.0 (GraphPad, San Diego, CA, USA) to evaluate statistical significance. A *p* value of < 0.05 was considered statistically significant for all the assays.

## 3. Results

### 3.1. Endogenous Ctp Is an Inner Membrane Protein

**Figure 1:**
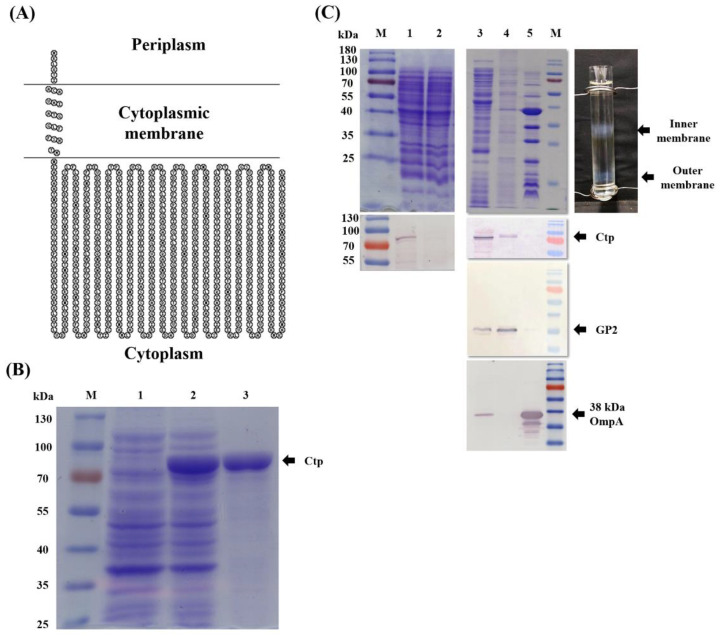
Ctp protein is localized in the cytoplasmic membrane. (**A**) A topology model for subcellular localization of Ctp, generated using the SACS MEMSAT server. (**B**) SDS-PAGE analysis showing M, marker; lanes 1 and 2, uninduced and IPTG-induced *E. coli* BL21 (DE3), respectively; and lane 3, Ni-NTA His-bind^®^ Resin affinity column purified Ctp protein. Cultures with and without IPTG were normalized to OD_600_ = 1, and wells were loaded with the same volume of lysate. (**C**) Top image shows SDS-PAGE analysis: lane 1, crude lysate from ATCC 17978; lane 2, crude lysate from MR14; and lanes 3, 4 and 5, cytoplasm, cytoplasmic membrane and OM proteins, respectively, obtained from ATCC 17978. Image on right shows separation of inner and outer membranes into distinct bands using sucrose gradient. Bottom image: Western blot analysis; each lane corresponds to the respective lane from the top image. Primary antibody of Ctp was used for the blotting assay. Antibodies specific to GP2 and 38 kDa OmpA were used as an internal loading control for inner and outer membrane fractions, respectively.

The N-terminal signal peptide prediction within the Ctp by SignalP-5.0 indicated that this protein is destined to cross the cytoplasmic membrane through the Sec-pathway [51]. The in silico (PSORTb 3.0.2) analysis also revealed that this protein was present in the cytoplasmic membrane. The SACS MEMSAT result showed that in the N-terminal of Ctp, amino acids (aa) 1–6 are located in the periplasmic region, residues from aa 7–23 form a transmembrane helix and the remaining C-terminal residues aa 24–727 are located in the cytosol (Figure 1A). To confirm the prediction, we overexpressed Ctp protein in *E. coli* and purified His-tagged protein to obtain an anti-Ctp polyclonal antiserum (Figure 1B). The subcellular localization of Ctp in *A. baumannii* was then identified through membrane fractionation and Western blotting with anti-Ctp as the primary antibody and goat antirabbit IgG-AP conjugate as the secondary antibody. The purity of membrane fractionation was confirmed by probing fractionated cells using inner and outer membrane specific antibodies namely GP2 and OmpA, respectively. As shown in Figure 1C, Ctp was lacking in the crude lysate of MR14 (lane 2), and the Ctp in *A. baumannii* was found to be localized in the cytoplasmic membrane (lane 4), consistent with the results obtained using bioinformatics tools. Probing of a PVDF membrane with GP2, an inner membrane specific antibody followed by reprobing with anti-Ctp polyclonal antiserum further helped us to know that endogenous Ctp is an inner membrane protein (Supplementary Figure S1).

### 3.2. Ctp Mutation Promotes CPS Production and Cell Aggregation

**Figure 2:**
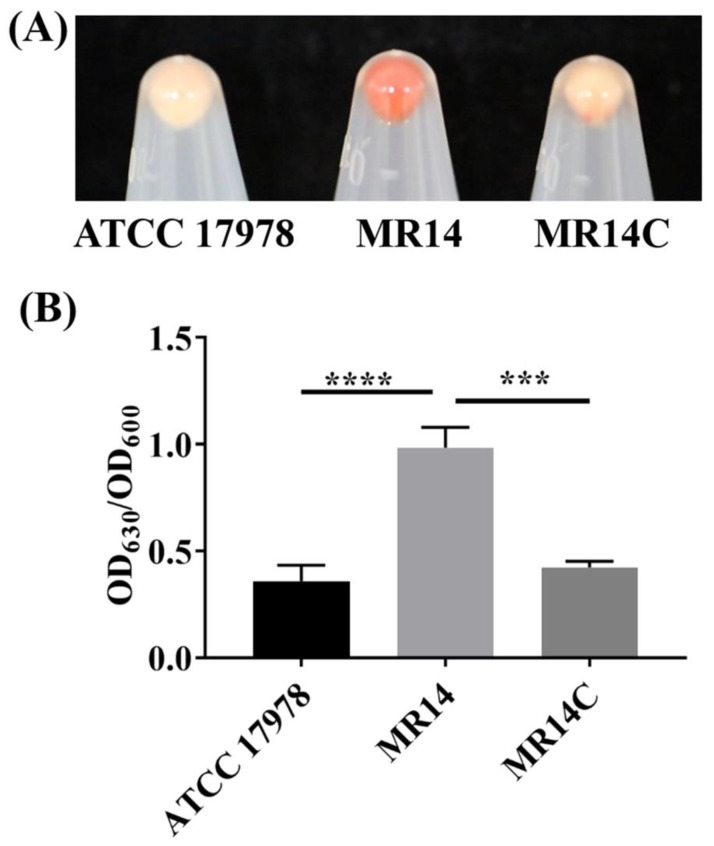
*ctp* mutation led to increased CPS production. (**A**) CPS visualization using CR binding of pellet cells. (**B**) CPS quantification using anthrone reagent. Data represent the mean ± SD of three independent experiments. *** *p* < 0.001, **** *p* < 0.0001.

In our previous study, we demonstrated MR14 to have a mucoidy and sticky phenotype [33]. In the present study, we evaluated the role of Ctp in CPS production. First, we examined the ability of *A. baumannii* strains to bind with CR. When the strains were grown in liquid LB containing CR, CR-bound aggregates of cells were discovered at the bottom and increased amounts of slime were attached to the wall of the culture glass tube for MR14 (Supplementary Figure S2A) compared with ATCC 17978 and MR14C, respectively. Upon centrifugation, MR14 showed greater CR staining (Figure 2A). Quantification of CPSs also demonstrated significantly more CPSs in MR14 compared with ATCC 17978 and MR14C (Figure 2B). Visualization of these strains under SEM revealed that MR14 was aggregated and covered with a polysaccharide-like structure (Supplementary Figure S2B). The low CPS production, low CR staining upon complementation of the *ctp* gene and SEM visualization confirm the role of Ctp in CPS production.

### 3.3. More Biofilm Matrix Surrounded ctp Mutant Due to Cell Content Release by Autolysis

**Figure 3:**
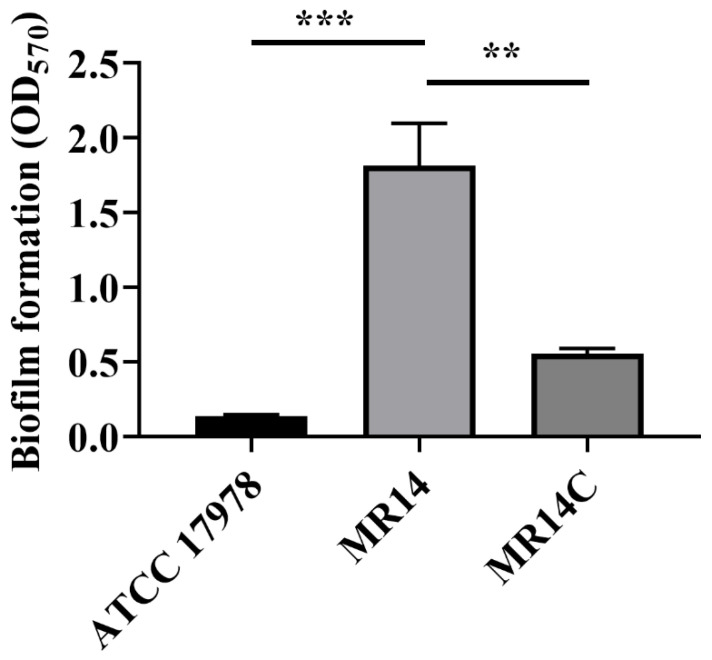
*ctp* mutation led to enhanced biofilm formation. Quantification of the biofilm formed by ATCC 17978, MR14 and MR14C using the crystal violet (CV) staining assay. *** *p* < 0.001 and ** *p* < 0.01, determined from three independent experiments, mean ± SD.

Several studies have reported that increased CPS content contributes to increased biofilm formation [52,53]. The quantitative analysis of the biofilm’s CV staining revealed that significantly more biofilm adhered to the glass surface in MR14 compared with ATCC 17978 and MR14C (Figure 3).

**Figure 4:**
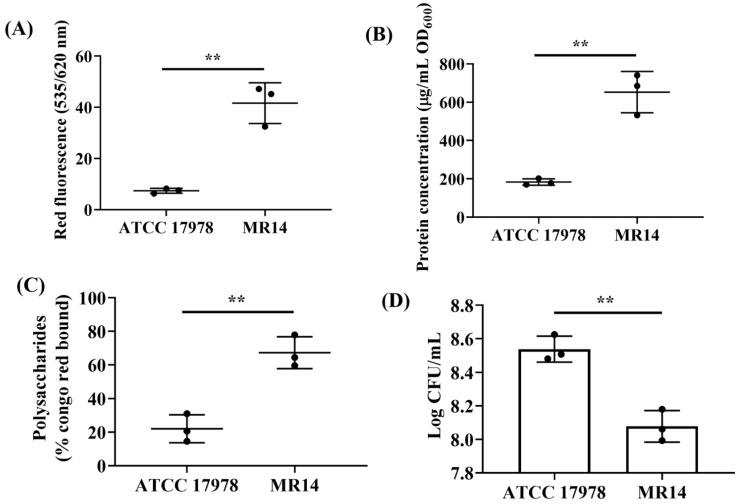
eDNA and proteins contribute to the biofilm phenotype of MR14. (**A**) eDNA was quantified in terms of fluorescence by using PI. (**B**) Amount of proteins present within the biofilm matrix of ATCC 17978 and MR14, quantified using a BCA assay. (**C**) Total polysaccharides of the biofilm matrix, quantified using the CR method. (**D**) Quantification of viable cells present within the biofilm of ATCC 17978 and MR14, obtained by serial dilution followed by spreading on agar plates. ** *p* < 0.01, determined from three independent experiments, mean ± SD.

The material released due to autolysis would either stay in culture medium or would contribute to growing biofilm by becoming its component or by contributing bacterial growth by acting as a source of nutrients. MR14 has been reported to undergo autolysis [33], hence we wanted to check if higher biofilm of MR14 is due to the higher amount of lysed cell’s product. We used PI to stain the eDNA in the cell-free supernatant obtained from the biofilms and observed higher fluorescence intensity in MR14 than in ATCC 17978 (Figure 4A). The biofilm of MR14 also contained significantly more proteins and polysaccharides (Figure 4B,C) compared with that of ATCC 17978. We also enumerated viable cells within the biofilms of ATCC 17978 and MR14. Despite the greater biofilm formation in MR14, the number of viable cells within the biofilm was found to be low (Figure 4D). The results suggest that the greater biofilm formation in MR14 was due to greater availability of components required for biofilm formation due to autolysis.

**Figure 5:**
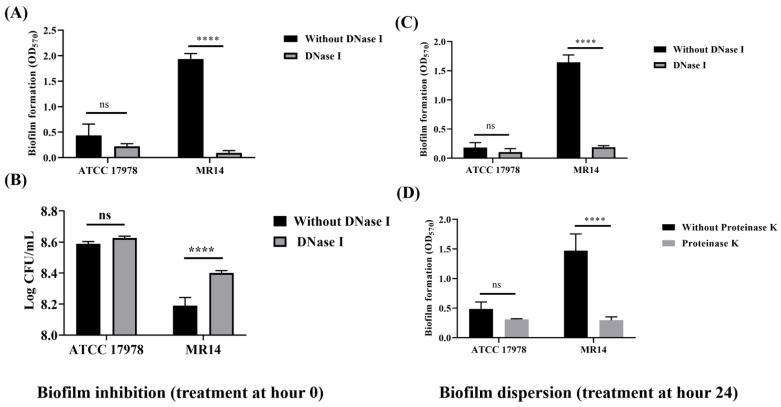
Enzymatic inhibition and disruption of biofilm formation in *A. baumannii*. (**A**) Inhibition assay, performed by adding DNase I at 0 h, allowing cultures to form biofilms for 24 h in the presence and absence of DNase I and performing quantification with CV. (**B**) Planktonic growth analysis conducted at 24 h during the biofilm inhibition. (**C**) and (**D**) Quantification of the biofilm after disruption of biofilm formation using DNase I and Proteinase K, respectively. **** *p* < 0.0001 and ns = no significant, determined using three independent cultures.

To further verify the contribution of eDNA and proteins to biofilm formation, we performed inhibition and dispersion biofilm assays. The addition of DNase I at 0 and 24 h, respectively, led to significantly less biofilm formation in MR14 compared with ATCC 17978 (Figure 5A,C). The results revealed that DNase I can inhibit biofilm formation and disperse the already formed biofilm in MR14. Greater planktonic growth in MR14 on the addition of DNase I at 0 h shows eDNA to be the key component of biofilm matrix that is necessary for initial attachment (Figure 5B). Since the cells that are meant to form a biofilm remained within the medium, the optical density OD_600_ was increased. Moreover, the addition of proteinase K at 24 h led to biofilm dispersion, as evidenced by a significant decrease in biofilm formation in MR14 (Figure 5D).

### 3.4. Microscopic Analysis of Biofilm Cells of ATCC 17978 and MR14

**Figure 6:**
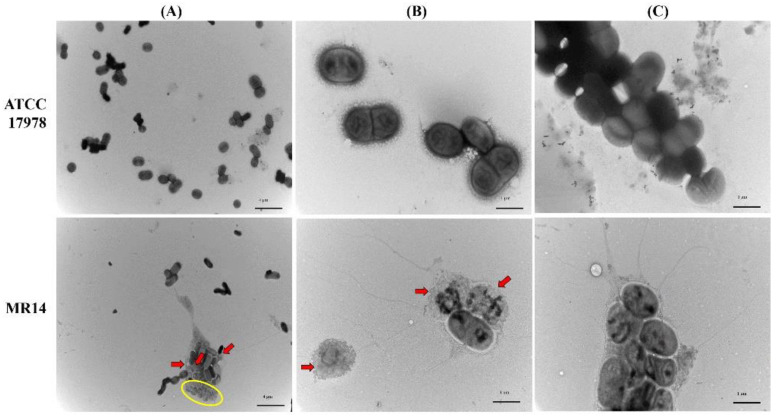
MR14 biofilm cells composed of numerous dead cells. TEM analysis of biofilm cells of ATCC 17978 (top images) and MR14 (bottom images). (**A**) TEM image at 5000× magnification. (**B**) and (**C**) TEM image at 20,000× magnification. Red arrows indicate lysed cells and ghost cells are encircled in yellow.

The bacterial cell morphology within the biofilm was characterized through TEM. Most of the cells in the biofilm of ATCC 17978 were found to be intact (Figure 6A–C). By contrast, the MR14 biofilm contained ghost cells with loss of cytoplasmic content but with intact cellular envelope, intact cells and lysed cells (Figure 6A), lysed cells and ghost cell (Figure 6B), and ghost cells that are aggregated with the products of the lysed cells and enclosed within a network of thread-like structures that was absent in the ATCC 17978 biofilm (Figure 6C). We also visualized vesiculation in biofilm cells and planktonic cells of ATCC 17978 and MR14 through TEM and observed the presence of membrane vehicles (MVs) in MR14 samples (Supplementary Figure S3). The MR14 cells undergoing lysis and visualization of the generation of MVs by the lysed cells suggest that OMV generation could also be the consequence of *ctp* mutation as previous study has reported cell lysis as one of the mechanism that contribute in production of MVs that includes OMVs and O-IMVs (outer-inner membrane vesicles) [54].

### 3.5. Ctp Mutant Is Sensitive to SDS and EDTA

**Figure 7:**
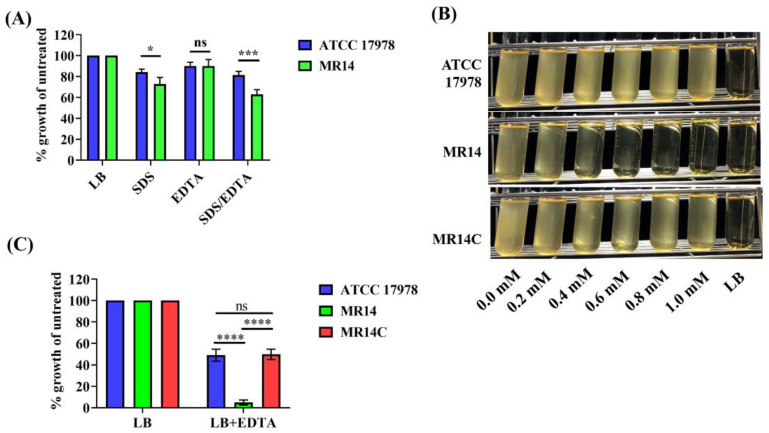
MR14 is sensitive to SDS and EDTA. (**A**) Percentage of growth, determined by measuring OD_600_ at 8 h in the presence of 0.01% SDS, 0.1 mM EDTA and SDS/EDTA compared with untreated samples. (**B**) Sensitivity of ATCC 17978, MR14 and MR14C to different concentrations of EDTA (0–1 mM). (**C**) Growth defect percentage as described in (**A**) in the presence of 0.4 mM EDTA alone. Data from *n* = 3 biological replicates are reported as mean ± SD. * *p* < 0.05, ns = not significant, *** *p* < 0.001 and **** *p* < 0.0001.

Our previous study showed that the *ctp* gene contributes to membrane integrity [33]. Furthermore, *A. baumannii zrlA* mutant was shown to have loss of OM integrity by assessing sensitivity of its planktonic cells to SDS/EDTA [47]. We anticipated that altered membrane integrity might cause the bacteria to be sensitive to membrane-permeabilizing agents such as SDS and EDTA. To confirm this hypothesis, we evaluated the sensitivity of planktonic cells of ATCC 17978 and MR14 to SDS, EDTA and SDS/EDTA and found that at 0.01% SDS, MR14 was more sensitive than ATCC 17978 (Figure 7A). The growth defects in ATCC 17978 and MR14 in the presence of 0.1 mM EDTA alone were not significant. However, when EDTA was combined with SDS, a more favorable synergetic effect was observed on the growth defect of MR14 compared with that of ATCC 17978. Moreover, MR14 was sensitive to increasing EDTA concentration as compared to wild-type ATCC 17978 and MR14C strain restored wild-type phenotype as shown by turbidity of LB that represent resistance to EDTA. (Figure 7B). EDTA alone (0.4 mM) could result in a significant growth defect of MR14 compared with ATCC 17978 and MR14C (Figure 7C). These results suggest that MR14 lysis is the consequence of OM permeability defects [55].

### 3.6. Ctp Mutation Causes Hypervesiculation Phenotype

**Figure 8:**
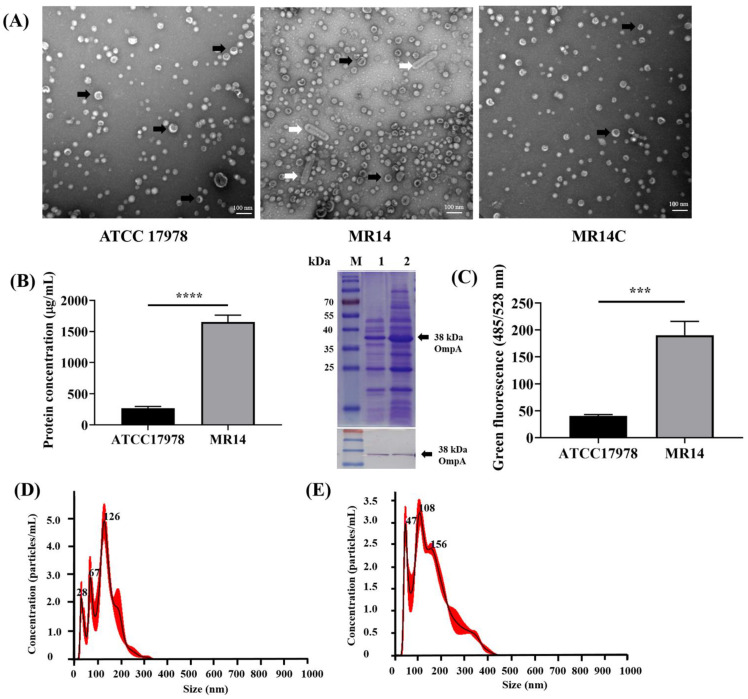
*ctp* mutation led to the hypervesiculation phenotype. (**A**) TEM analysis of membrane vesicles obtained from ATCC 17978, MR14 and MR14C. OMVs and OMTs are indicated by black and white arrows, respectively. (**B**) Left graph: OMV quantification by using the BCA method; right image: results of the SDS-PAGE analysis of OMVs obtained from ATCC 17978 and MR14; and bottom image: results of the Western blotting analysis using an antibody against 38-kDa OmpA. (**C**) Quantification of eDNA associated with OMVs of ATCC 17978 and MR14. (**D**) and (**E**) Histograms of size and particle distribution obtained using nanoparticle tracking analysis of OMVs isolated from ATCC 17978 and MR14, respectively. Graphs represent the mean ± SD of three independent replicates. *** *p* < 0.001 and **** *p* < 0.0001.

Due to the lowered membrane integrity, we examined the effect of *ctp* gene mutation on OMV biogenesis. We performed a TEM analysis of ATCC 17978, MR14 and MR14C strains isolated at different time points, namely at 3, 5, 12 and 18 h. We observed blebbed, OMV-like structures around the MR14 cells, which have rarely been seen in ATCC 17978 and MR14C cells (Supplementary Figure S4). To further verify these observations, we isolated the OMVs, visualized them through TEM and quantified them using BCA assay (Pierce BCA protein assay kit). As expected, we observed more MR14 OMVs than ATCC 17978 and MR14C OMVs (Figure 8A). Along with OMVs, MR14 was found to generate outer membrane tube (OMT)-like structures. A study on *Francisella novicida* reported the secretion of similar OMT-like structures along with OMVs [56]. The higher OMV levels in MR14 were further quantified in terms of protein concentration (Figure 8B). The quantification was followed by SDS-PAGE analysis of the OMVs obtained from ATCC 17978 and MR14. The results clearly indicated higher protein concentrations in MR14 OMVs than in ATCC 17978 OMVs (Figure 8B). The SDS-PAGE analysis revealed protein bands similar to bands of total OM protein obtained from ATCC 17978 reported in another study [57], further confirming that the vesicles generated were derived from the OM. Along with higher protein concentrations, we observed significantly higher nucleic acid concentrations associated with MR14 OMVs compared with ATCC 17978 OMVs, as measured using fluorescence (Figure 8C). An NTA analysis showed that the OMV samples from ATCC 17978 and MR14, when normalized to the same protein concentration of 1 mg/mL, contained 4.63 × 10^11^ and 5.13 × 10^11^ particles/mL, respectively (Figure 8D,E). The mean size of the OMVs of ATCC 17978 and MR14 was 125.7 and 159.6 nm, respectively.

### 3.7. OMVs from ctp Mutant Are More Virulent Than Parental Strain

**Figure 9:**
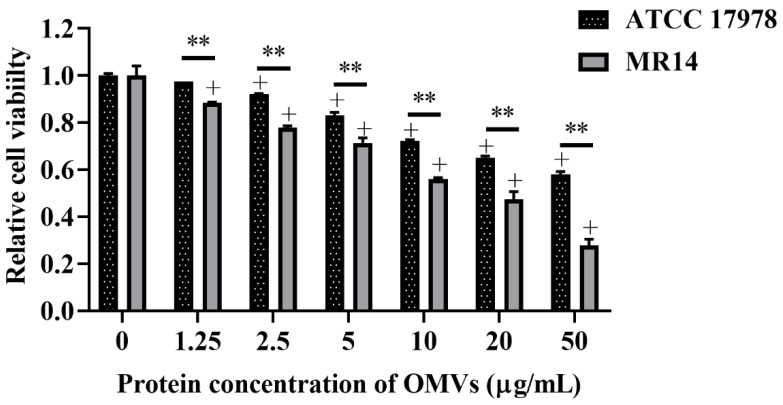
*ctp* mutation increases OMV-mediated host cell cytotoxicity. The host cell cytotoxicity was measured by treating A549 cells with different concentrations of OMVs isolated from ATCC 17978 and MR14 for 24 h and then performing an MTT assay. Data are presented as mean ± SD of three independent experiments + *p* < 0.05 compared with untreated control. ** *p* < 0.01 for comparison of cytotoxicity at the same concentration of OMVs between ATCC 17978 and MR14.

The OMVs isolated from *A. baumannii* have been reported to possess cytotoxic activity against the host cell [50]. Therefore, we determined the host cell cytotoxicity induced by OMVs isolated from ATCC 17978 and MR14. Compared with the untreated control, the OMVs from ATCC 17978 triggered cytotoxicity at concentrations ≥2.5 µg/mL. By contrast, OMVs from MR14 were observed to trigger cytotoxicity at concentrations ≥1.25 µg/mL. The cytotoxicity of OMVs from ATCC 17978 and MR14 significantly differed at concentrations ≥1.25 μg/mL (Figure 9). This result suggested that *ctp* mutation may pack more OM materials and periplasmic proteins into OMVs due to the loss of membrane integrity; consequently, the OMVs produced from MR14 were not only larger in size but also more toxic than those from ATCC 17978.

## 4. Discussion

The current study is an extension of our previous research [33]. This study provides additional insights into the Ctp of *A. baumannii* and the fate of *ctp* mutant. Localization studies investigating Prc protease have shown that it is present in the periplasm and cytoplasmic membrane in *E. coli* [58], only in the periplasm in *P. aeruginosa* [51] and in the cell wall of *Staphylococcus aureus* [59]. In our study, Ctp was found in the cytoplasm and cytoplasmic membrane fractions. Diversity in gene sequences among different species may be responsible for the difference in subcellular locations and substrate specificity, thus imparting unique functions in each bacterial species.

A positive correlation was observed between lysis of a subpopulation of cells and biofilm formation. eDNA is released as the product of cell lysis into the culture medium and can impart various functions to the cells. eDNA has been shown to increase the hydrophobicity of bacterial cell surfaces and be critical for adhesion to glass surface [60]. A report on clinical strains of *A. baumannii* showed that the degree of hydrophobicity is directly proportional to adhesion to the abiotic surface [61]. Furthermore, a study on clinical isolates of *A. baumannii* in International Clone Lineage II demonstrated higher biofilm-forming ability of strains that are more hydrophobic compared with hydrophilic strains [62]. In our previous study, we observed that MR14 was significantly more hydrophobic than ATCC 17978 and released significantly higher amounts of eDNA into the culture medium [33]. In the present study, we found that autolysis of MR14 contributes to eDNA and protein release, which promotes biofilm formation. Increased eDNA might also have caused MR14 to become more hydrophobic, thus promoting biofilm formation. Our results are in accordance with those of a previous study that demonstrated increased eDNA in the *clpP* mutant and subsequently increased biofilm formation as a consequence of increased activity of Sle1, a PG hydrolase [63]. A previous study reported a result similar to that of ours, wherein *Klebsiella pneumoniae* with high biofilm-formation ability had numerous dead cells and a few live cells and *K. pneumoniae* with low biofilm-formation ability had numerous live cells and a few dead cells [44]. We expect that the underlying mechanism is the same. In contrast to our observations, *ctpA* function was reported to be necessary for biofilm accumulation and maturation in *R. leguminosarum* [64]. This difference in biofilm phenotype is expected to be attributed to the difference in cell death rate and availability of eDNA that modifies cell surface properties and facilitates cell interaction with the abiotic surface through acid–base interaction [65]. Furthermore, we demonstrated more eDNA on the surface of MR14 OMVs. Previous studies have confirmed the presence of OMV-associated DNA within the biofilm of gram-negative and gram-positive bacteria [66,67], and another study demonstrated that OMVs secreted by *P. putida* DOT-T1E had enhanced bacterial cell surface hydrophobicity and biofilm formation [68]. In general, high biofilm-formation ability is positively associated with virulence, but the results of our previous study showed that MR14 significantly reduced virulence in zebrafish and reduced the adherence to and invasion of A549 cells [33]. In the present study, we demonstrated that the higher ability of MR14 to form biofilms was due to eDNA and proteins release induced by autolysis rather than the bacteria themselves. This could be why the higher biofilm-forming ability of MR14 does not corroborate the virulence shown by the MR14 cells.

It is evident from a previous study on *E. coli* that deletion of *prc* protease leads to leakage of periplasmic proteins due to higher OM permeability [58]. The higher sensitivity of MR14 to SDS and EDTA is attributed to the loss of OM integrity. Our result is in accordance with a previous study that demonstrated reduced growth of the *tsp* mutant in the presence of SDS and EDTA [69]. We believe that MR14 autolysis is likely due to loss of OM integrity caused by dysregulation of PG dynamics. The increasing emergence of antibiotic resistance among *A. baumannii* has resulted in therapeutic challenges, especially in hospital-acquired infections. In such scenarios, the use of certain drugs or inhibitors to inhibit *ctp* gene function makes *A*. *baumannii* more susceptible to reagents containing EDTA and SDS, which may prevent its growth and colonization in hospital environments and on equipment.

OMVs generated by the hypervesiculating MR14 exhibited higher cytotoxicity toward A549 cells. This result is consistent with that of previous studies showing that OMVs from hypervesiculating ATCC 17978 mutants exhibit increased cytotoxicity toward A549 cells [50,70]. *A. baumannii* has been reported to secrete cytotoxic OmpA through OMVs [71]. In addition, OmpA-deficient *A. baumannii* OMVs have shown reduced ability to induce cell death of J774 mice macrophages and A549 cells [72]. Furthermore, in a previous study, ATCC 17978 *bfmS* deletion mutant showed hypervesiculation phenotype and the OMVs derived from it displayed more OmpA and greater cytotoxicity toward A549 cells compare with wild-type OMVs [50]. Since OmpA is the most abundant protein in *A. baumannii* [73], any change in the OMV size distribution as shown using NTA could contributes to an increase in the OmpA level in MR14 OMVs. The western blot analysis as shown in Figure 8B revealed that OmpA profile were similar between OMVs from ATCC 17978 and MR14. Even though western blot result shows similar protein level, difference in protein content can’t be excluded as suggested by previous study [70], that SDS-PAGE analysis is less appropriate than two-dimensional gel electrophoresis. However, in this study we didn’t investigate the OMVs from ATCC 17978 and MR14 in terms of cytotoxic factors and protein content and further experiments are required to verify the underlying mechanism behind higher cytotoxicity of MR14 OMVs compared to ATCC 17978 OMVs. As shown in Supplementary Figure S4, prior to undergoing autolysis, MR14 cells were observed to produce more OMVs compared with ATCC 17978 and MR14C cells. In addition, MVs were shown to be generated by MR14 cells undergoing autolysis potentially in a manner described previously [15]. Autolysis was not observed in ATCC 17978. From these observations, we conclude that MR14 can generate OMVs both in an autolysis-dependent and -independent manner and that *ctp* mutation first leads to hypervesiculation, followed by excessive secretion of OMVs and loss of cell membrane integrity and cell envelope homeostasis. Finally, the cells burst and undergo lysis, producing more MVs along with eDNA and proteins required for biofilm formation. The hypervesiculation phenotype upon *ctp* gene mutation in *A. baumannii* could be the result of the improper PG biosynthesis caused by the altered activity of autolysins, cell wall hydrolases and aggregation of misfolded periplasmic proteins as mentioned in the introduction. We believe that MR14 autolysis contributed to the secretion of eDNA into the culture medium, with this eDNA possibly becoming attached to the surface of MR14 OMVs, as shown in Figure 8C, potentially by linking to an eDNA/OMV network. To our knowledge, few reports have mentioned the role of *ctp* in *A. baumannii* and this is the first report to demonstrate the hypervesiculation phenotype of the ATCC 17978 *ctp* mutant.

Previous studies on *zrlA* have mentioned that this gene in *A. baumannii* exhibited peptidase activity such as that of *spr* in *E. coli*, and properties of the *zrlA* mutant such as loss of membrane integrity, sensitivity to SDS/EDTA, motility defects, reduced adherence to and invasion of A549 cells, membrane localization and overproduction of OMVs causing more cytotoxicity toward A549 cells are similar to the properties of MR14 [33,47,70,74]. Since Spr in *E. coli* is known to be the substrate of Prc protease [75], the aforementioned properties led us to hypothesize that *ctp* directly or indirectly regulates ZrlA, a cell wall modifying enzyme in *A. baumannii*. However, further experiments are needed to verify this hypothesis. The only property of the *zrlA* mutant that differs from that of MR14 is the lower biofilm-forming ability compared with ATCC 17978. This could be because of the difference in viability defects because the *zrlA* mutant showed no growth defects or autolysis [70]. The difference in biofilm phenotype can be attributed to the fact that Ctp can target multiple cell wall hydrolases [10], and the viability defects could be attributed to the multiple substrates dysregulation. On the basis of our current findings, we speculate that the combination of specific peptide inhibitors against Ctp with DNase I or EDTA could pave the way for developing novel therapeutics against infections caused by *A. baumannii*. Since MR14 overproduces OMVs, these OMVs without or with some remodeling (e.g., optimizing the immune response) can be explored for the development of broad-spectrum OMV-based vaccines against *A. baumannii*.

## 5. Conclusions

Taken together, the study findings elucidate the consequences of *ctp* gene mutation in term of OMV biogenesis and biofilm formation in *A. baumannii*. We found that Ctp is located in the cytoplasmic membrane of *A. baumannii* and that the higher biofilm-forming ability of MR14 is the result of the release of eDNA and protein caused by cell lysis. Our results indicated that MR14 exhibits a hypervesiculation phenotype, the production of vesicles could be the indirect effect of elevated cell envelope stress responses and enhanced membrane defect due to loss of Ctp, and the OMVs produced by MR14 are more cytotoxic to the host cell than are ATCC 17978 OMVs. These results indicate the importance of Ctp in the integrity of *A. baumannii* cell membranes and controlling virulence by undergoing hypervesiculation by cells lacking Ctp.

## Data Availability

Not applicable.

## Supplementary Materials

The following are available online at https://www.mdpi.com/article/10.3390/microorganisms9061336/s1. Figure S1: Subcellular localization of Ctp in *A. baumannii*; Figure S2: CPSs visualization using CR and SEM; Figure S3: TEM analysis of biofilm and planktonic cell growth; Figure S4: *ctp* mutation led to hypervesiculation, morphological defects and autolysis.

## Abbreviations

Ctp	Carboxy-terminal processing protease
ATCC	American Type Culture Collection
eDNA	Extracellular DNA
SDS	Sodium dodecyl sulfate
EDTA	Ethylenediaminetetraacetic acid
OMVs	Outer membrane vesicles
PG	Peptidoglycan
PBPs	Penicillin-binding proteins
OM	Outer membrane
PCR	Polymerase chain reaction
LB	Luria broth
OD	Optical density
PBS	Phosphate-buffered saline
RT	Room temperature
BCA	Bicinchoninic acid
CPSs	Capsular polysaccharides
CR	Congo Red
CV	Crystal violet
PI	Propidium iodide
CFUs	Colony forming units
NTA	Nanoparticle tracking analysis
MTT	3-(4,5-dimethylthiazol-2-yl)-2,5-diphenyl tetrazolium bromide
OMT	Outer membrane tubes
MVs	Membrane vesicles
