# Supplementary material for: Carboxy-Terminal Processing Protease Controls Production of Outer Membrane Vesicles and Biofilm in Acinetobacter baumannii
Source: Microorganisms. 2021 Jun 20;9(6):1336. doi: 10.3390/microorganisms9061336 (PMC8234194; doi:10.3390/microorganisms9061336)
Supplement: Supplementary file 1 [file microorganisms-09-01336-s001.zip › microorganisms-1224280-supplementary.pdf]

microorganisms

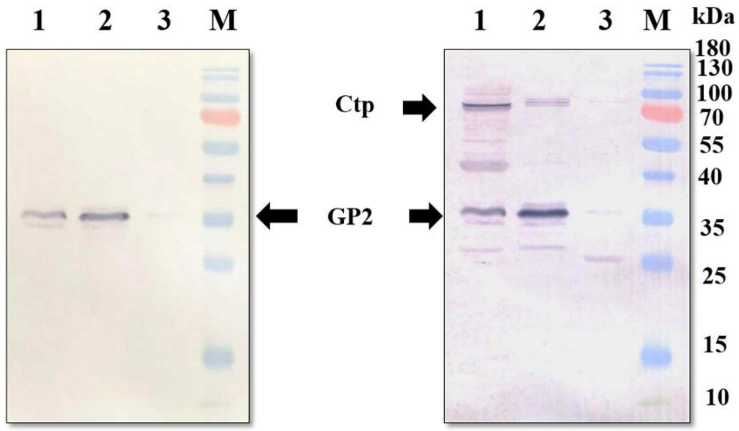

Supplementary figure S1. Subcellular localization of Ctp in *A. baumannii*. lanes 1, 2 and 3, represents cytoplasm, cytoplasmic membrane and OM proteins, respectively, obtained from ATCC 17978. Image on the left shows western blot image blotted with GP2, an inner membrane specific antibody and image on the right shows reprobing of PVDF membrane on left with anti-Ctp polyclonal antiserum.

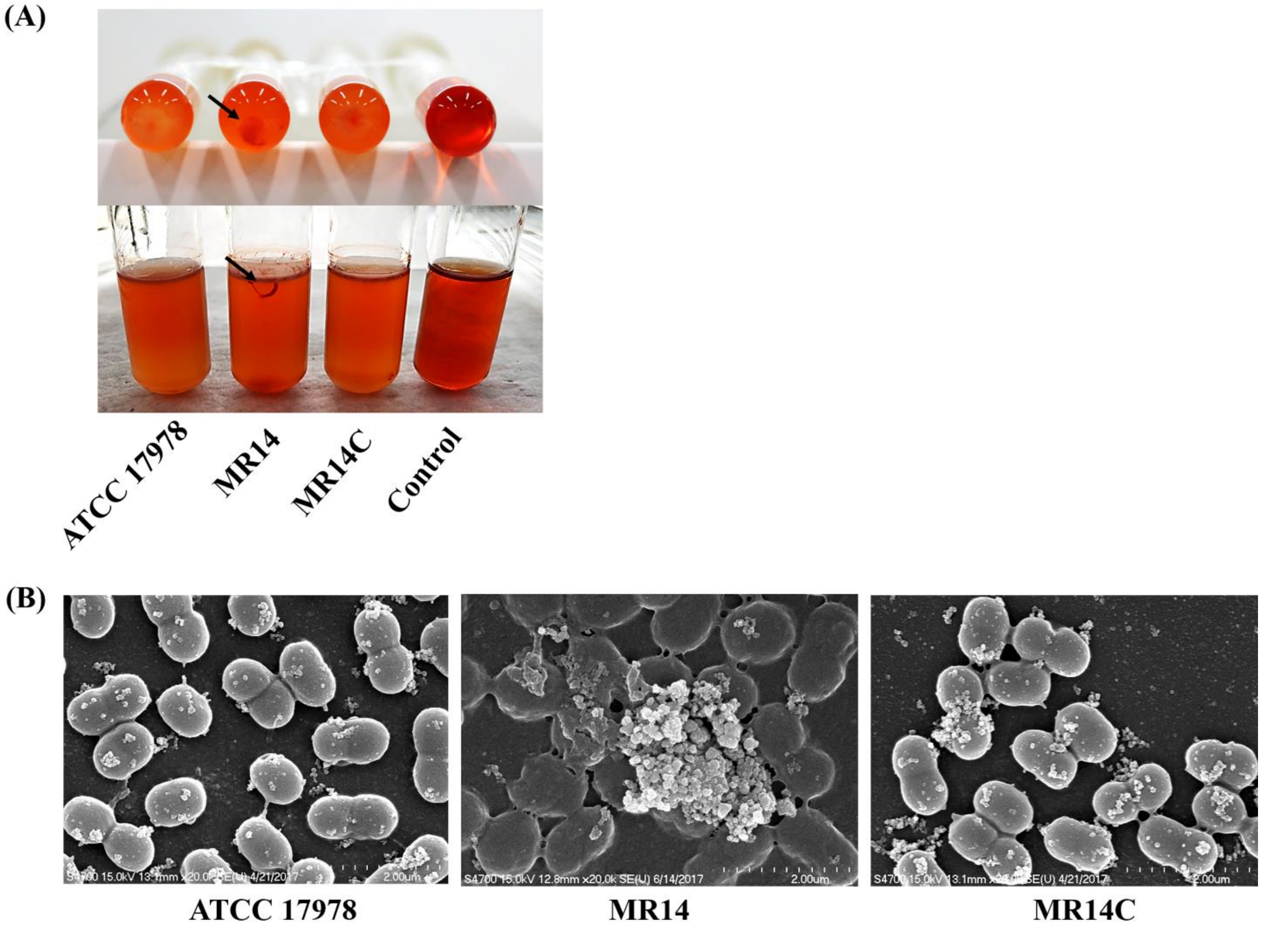

Supplementary figure S2. CPSs visualization using CR and SEM. (**A**) Top image: CR-bound aggregates at the bottom of the tube. Bottom image: slime production at the air-liquid interface on the glass surface, denoted by arrows. Left to right ATCC 17978, MR14, MR14C, and Control (LB-CR). (**B**) SEM image showing aggregation and CPS-like structure production in MR14.

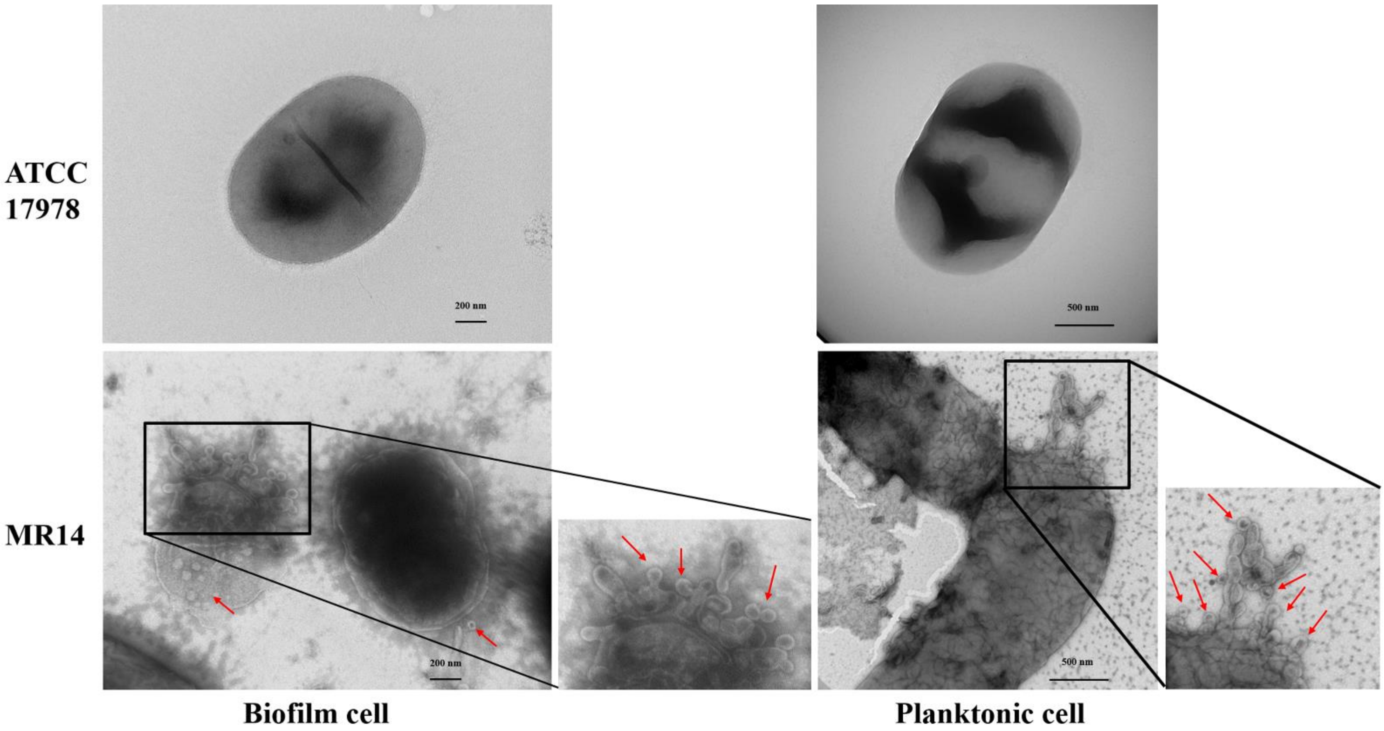

Supplementary figure S3. TEM analysis of biofilm and planktonic cell growth. Top image: biofilm cell (left) and planktonic cell (right) of ATCC 17978. The cells appear healthy and intact. Bottom image: biofilm cell (left) and planktonic cell (right) of MR14. In contrast to those of ATCC 17978, both the biofilm and planktonic cell of MR14 produced MVs (shown by red arrows) either through autolysis or shedding.

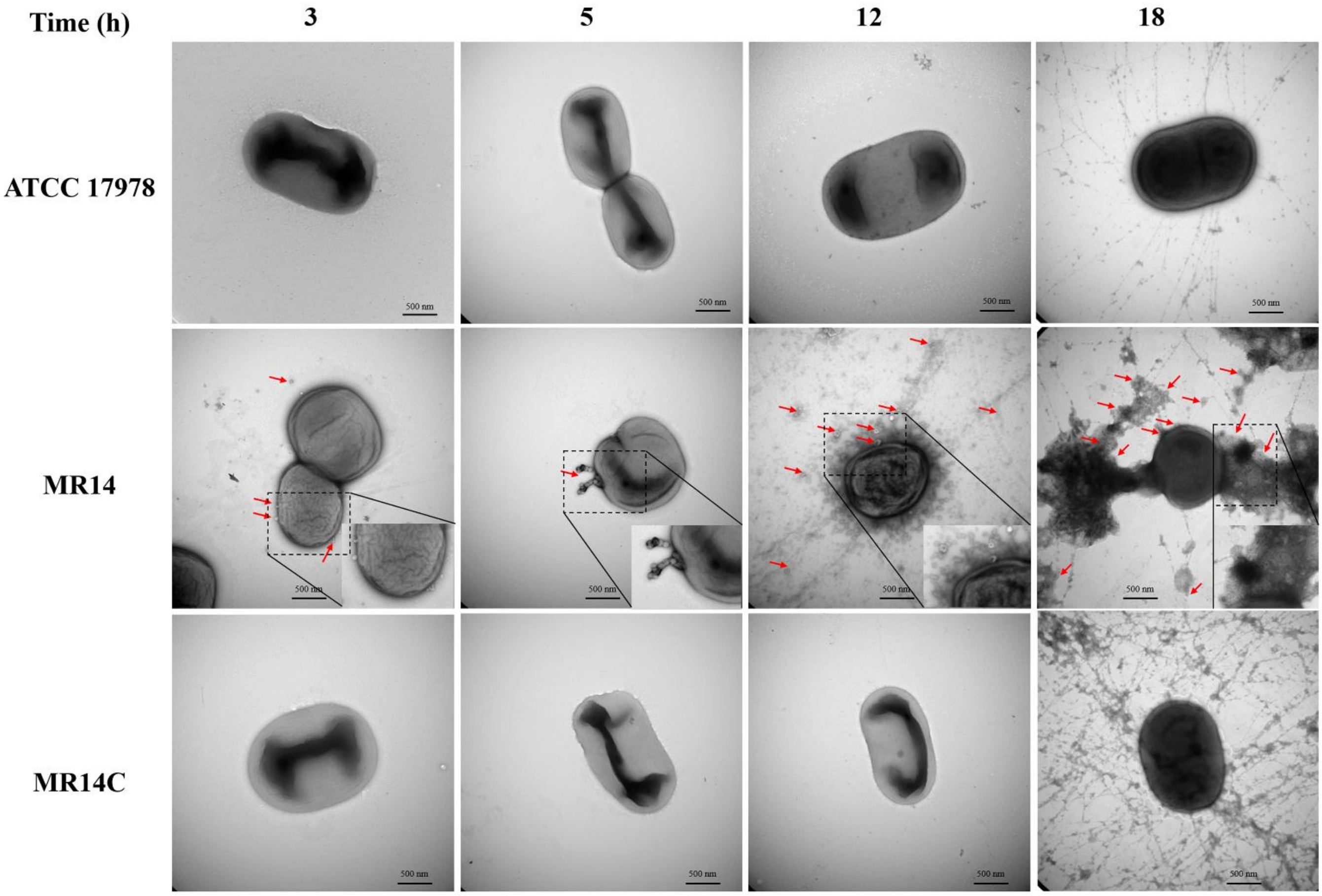

Supplementary figure S4. *ctp* mutation led to hypervesiculation, morphological defects, and autolysis. Compared with ATCC 17978 and MR14C, MR14 showed blebbing at 3 h, morphological defects along with blebbing and OMVs around the cell at 5 and 12 h, respectively, and cell lysis and production of MVs as the lysed cell products at 18 h. Blebbing and the MVs are indicated by red arrows.
